# Supplementary material for: Positive Effects of an Online Workplace Exercise Intervention during the COVID-19 Pandemic on Quality of Life Perception in Computer Workers: A Quasi-Experimental Study Design
Source: Int J Environ Res Public Health. 2022 Mar 7;19(5):3142. doi: 10.3390/ijerph19053142 (PMC8910608; doi:10.3390/ijerph19053142)
Supplement: Supplementary file 1 [file ijerph-19-03142-s001.zip › ijerph-1578986-supplementary/File S1_Questionnaire.pdf]

Article

Positive effects of an online workplace exercise intervention during the COVID-19 pandemic, on quality of life perception in Computer Workers: A quasi-experimental study design

Sara Moreira* [1,2,3], Maria Begoña Criado[3,4], Maria Salomé M Ferreira[2,5], Jorge Machado [1,3,6], Carla Gonçalves[7,8], Filipe Manuel Clemente[7,8,9], Cristina Mesquita [10,11], Sofia Lopes [10,11,12] and Paula Clara Santos [10,11,13]

CÓDIGO ☐

Data ___/___/___

Parte I – Questionário Sócio-demográfico

A – Características Gerais

1. Qual é a sua data de nascimento? _____/_____/_____ **2.** Qual o seu género? ☐ feminino ☐ masculino

2. Qual é a sua altura? ______________ cm **3.** Qual é o seu peso actual? _______ Kg

5. Qual é o seu estado civil? ☐ solteiro ☐ casado/união de facto ☐ viúvo ☐ separado/divorciado

6. Habilitações literárias:

6.1. Quais são as suas habilitações literárias?

☐ 1ºCiclo ☐ 2ºCiclo ☐ 3ºCiclo ☐ Licenciatura ☐ Mestrado ☐ Doutoramento

7. Atividade Laboral:

7.1. Qual é o seu posto de trabalho? ____

7.2. Quantas horas por dia trabalha no seu posto de trabalho? _______horas/dia

7.3 Trabalha por turnos? ☐ sim (passe para a questão 7.4) ☐ não (passe para a questão 8)

7.4 Qual o seu turno de trabalho? ☐ manhã ☐ tarde ☐ noite

7.5 O seu turno é rotativo em horário? ☐ sim ☐ não

7.6 Qual o número de anos de serviço? _______ anos.

8. Exerce outra atividade laboral remumerada, para além desta? ☐ sim ☐ não

8.1 **Se sim,** quantas horas por semana despende nessa atividade? _______horas/semana

9. Situação em relação ao emprego:

9.1. Qual é a sua situação atual em relação ao emprego?

☐ Pertence ao quadro de pessoal da empresa ☐ Contrato individual de trabalho

☐ Outra (exemplo: licença sem vencimento) Qual? _______________________

10. Atualmente exerce funções de direção; chefia ou coordenação? ☐ sim ☐ não

11. Em media, quanto tempo **fora do horário** de trabalho, gasta semanalmente em **tarefas profissionais**:

11.1 Horas extraordinárias no local de trabalho_______horas/semana

11.2 Horas de trabalho extra realizadas em casa_______horas/semana

12. Em media, quanto tempo fora do horário de trabalho, gasta semanalmente em:

12.1 atividades domésticas (arrumar a casa; tratar da roupa, etc) _______hora/semana

12.2 atividades de lazer (ler, passear, estar no café com amigo a conviver etc) _______horas/semana

13. Atividade Laboral a partir de Março 2020, pós implementação de plano contingencia consequencia da COVID-19.

13.1 Trabalha por clusters? ☐ sim (passe para a questão 13.1.2) ☐ não (passe para a questão 13.2)

13.1.2 Qual o seu cluster de trabalho? ☐A ☐ B ☐ C

13.2 Tem experiencia em teletrabalho anterior a Março de 2020? ☐ sim (passe para a questão 13.2.1) ☐ não (passe para a questão 13.3)

13.2.1 Se sim com que frequência? ☐ 1 a 2x/semana ☐ 2 a 3x/semana ☐ + de 4x/semana

13.3 Semanalmente, com que frequencia está em modo presencial na empresa?

☐ Nunca ☐ 1 a 2x/semana ☐ 2 a 3x/semana ☐ + de 4x/semana ☐ diariamente (5x/semana)

13.4 No regresso a normalidade, pós-COVID-19, gostaria que p teletrabalho fizesse parte da sua rotina profissional?

☐ Nunca ☐ 1 a 2x/semana ☐ 2 a 3x/semana ☐ + de 4x/semana ☐ diariamente (5x/semana) ☐ não se aplica ao desempenho da minha tarefa.

B – Características do estado geral de Saúde

13. É portador de alguma doença diagnosticada? ☐ sim ☐ não

13.1. Se sim, qual? ______________

14. Alguma vez esteve de baixa médica por motivos de doença, acidente ou lesão? ☐ sim ☐ não

14.1. Se sim, quanto tempo aproximadamente? ________ dias, meses, anos (riscar o que não interessa)

14.2. Se sim, qual o motivo?

☐ Lesão, acidente ou doença decorrente da sua atividade laboral. Qual? _____________

☐ Assistência a outrém (exemplo: familiar)

☐ Lesão acidente ou doença decorrente **fora** atividade laboral. Qual? ____________

Parte II - Questionário de Avaliação de Qualidade de Vida Relacionada com a Saúde

(Medical Outcomes Study 36-Item Short Form Health Survey- MOS - SF- 36)

CÓDIGO [ ]

Este questionário visa fornecer-nos informações sobre a sua saúde e o seu nível de bem-estar nas **atividades da vida diária**. Responda a cada questão marcando a resposta como se indica na própria pergunta. Caso se sinta insegura sobre a melhor opção para responder, por favor, tente responder o melhor que lhe for possível.

1. **Em geral**, diria que a sua saúde é: *(coloque um circulo à volta de um número)*

Excelente	1
Muito boa	2
Boa	3
Ruim	4
Muito ruim	5

2. **Comparada com a sua saúde há um ano atrás**, como classificaria **agora** a sua saúde em geral: (*coloque um circulo à volta de um número*)

Muito melhor agora que há um ano atrás	1
Um pouco melhor agora do que há um ano atrás	2
Quase a mesma de há um ano atrás	3
Um pouco pior agora do que há um ano atrás	4
Muito pior agora do que há um ano atrás	5

3. Os itens seguintes são sobre atividades que poderia fazer atualmente durante um dia típico. **Atualmente, devido à sua saúde tem dificuldade** em fazer essas atividades? Se sim, quanto? (*coloque um circulo à volta de um número em cada linha*)

Atividades	Muita dificuldade	Dificulta um pouco	Não dificulta nada
a. **Atividades vigorosas**, que exigem muito esforço (correr, levantar objetos pesados, participar em desportos árduos, etc.)	1	2	3
b. **Atividades moderadas**, tais como mover uma mesa, usar o aspirador de limpeza, jogar à bola ou varrer a casa	1	2	3
c. Levantar ou carregar mantimentos	1	2	3
d. Subir **vários** lances de escada	1	2	3
e. Subir **um** lance de escada	1	2	3
f. Curvar-se, ajoelhar-se ou dobrar-se	1	2	3
g. Andar **mais de um quilómetro**	1	2	3
h. Andar **vários quarteirões**	1	2	3
i. Andar **um quarteirão**	1	2	3
j. Tomar banho ou vestir-se	1	2	3

4. **Durante as últimas 4 semanas,** teve alguns dos seguintes problemas com o seu trabalho ou com outra atividade diária regular, **como consequência da sua saúde física**? (*coloque um circulo à volta de um número em cada linha*)

	Sim	Não
a. Diminuiu a **quantidade de tempo** que se dedicava ao seu trabalho ou a outras atividades?	1	2
b. **Realizou menos tarefas** do que gostaria de ter realizado?	1	2
c. Esteve limitada no seu **tipo** de trabalho ou em outras atividades?	1	2
d. Teve dificuldade em realizar o seu trabalho ou outras atividades (por exemplo, necessitou fazer um esforço extra)?	1	2

5. **Durante as últimas 4 semanas**, teve algum dos seguintes problemas com o seu trabalho ou outra atividade diária regular, **como consequência de algum problema emocional** (como sentir-se deprimida ou ansiosa)? (*coloque um circulo à volta de um número em cada linha*)

	Sim	Não
a. Diminuiu a **quantidade de tempo** que se dedicava ao seu trabalho ou a outras atividades?	1	2
b. **Realizou menos tarefas** do que gostaria de ter realizado?	1	2

c. Não trabalhou ou não fez qualquer uma das outras actividades com tanto **cuidado** como geralmente faz?	1	2

6. **Durante as últimas 4 semanas**, de que maneira a sua saúde física ou problemas emocionais interferiram nas suas actividades sociais normais, em relação à família, vizinhos, amigos? (*coloque um circulo à volta de um número*)

De forma nenhuma	Ligeiramente	Moderadamente	Bastante	Extremamente
1	2	3	4	5

7. Quanta dor no corpo teve **durante as últimas 4 semanas**? *(coloque um circulo à volta de um número)*

Nenhuma	Muito leve	Leve	Moderadamente	Grave	Muito grave
1	2	3	4	5	6

8. **Durante as últimas quatro semanas**, em que medida é que a dor interferiu com o seu trabalho normal (incluindo tanto o trabalho fora de casa como em casa)? (*coloque um circulo à volta de um número*)

De maneira nenhuma	Um pouco	Moderadamente	Bastante	Extremamente
1	2	3	4	5

9. Estas questões são sobre como se sente e como tudo tem acontecido consigo durante as **últimas 4 semanas**. Para cada questão, por favor, dê a resposta que mais se aproxima da maneira como se sente em relação às últimas 4 semanas. (*coloque um circulo à volta de um número em cada linha*)

	Todo o tempo	A maior parte do tempo	Uma boa parte do tempo	Alguma parte do tempo	Uma pequena parte do tempo	Nunca
a. Quanto tempo se tem sentido cheia de vigor, cheia de vontade, cheia de força?	1	2	3	4	5	6
b. Quanto tempo se tem sentido uma pessoa muito nervosa?	1	2	3	4	5	6
c. Quanto tempo se tem sentido tão deprimida que nada pode animá-la?	1	2	3	4	5	6
d. Quanto tempo se tem sentido calma e tranquila?	1	2	3	4	5	6
e. Quanto tempo se tem sentido com muita energia?	1	2	3	4	5	6
f. Quanto tempo se tem sentido desanimada e abatida?	1	2	3	4	5	6
g. Quanto tempo se tem sentido esgotada?	1	2	3	4	5	6
h. Quanto tempo se tem sentido uma pessoa feliz?	1	2	3	4	5	6
i. Quanto tempo se tem sentido cansada?	1	2	3	4	5	6

10. **Durante as últimas quatro semanas**, quanto tempo é que a sua **saúde física** ou **problemas emocionais** interferiram com as suas atividades sociais (como visitar amigos, parentes, etc.)?
(*coloque um circulo à volta de um número*)

Todo o tempo	A maior parte do tempo	Alguma parte do tempo	Uma pequena parte do tempo	Nenhuma parte do tempo
1	2	3	4	5

11. Em que medida cada uma das frases seguintes é **verdadeira** ou **falsa** para si?
(*coloque um circulo à volta de um número em cada linha*)

	Definitivamente verdadeiro	A maioria das vezes verdadeiro	Não sei	A maioria das vezes falso	Definitivamente falso
a. Eu costumo adoecer um pouco mais facilmente que as outras pessoas	1	2	3	4	5
b. Eu sou tão saudável quanto qualquer pessoa que conheço	1	2	3	4	5
c. Eu acho que a minha saúde vai piorar	1	2	3	4	5
d. A minha saúde é excelente	1	2	3	4	5
